# Quality of Life after Transcatheter Aortic Valve Replacement in Sarcopenic Patients Using the Toronto Aortic Stenosis Quality of Life Questionnaire

**DOI:** 10.3390/jcm12052078

**Published:** 2023-03-06

**Authors:** Gabriele Pesarini, Alessandro Ruzzarin, Matteo Bonatti, Felix Pescoller, Patrick Engl, Rainer Oberhollenzer, Flavio Luciano Ribichini, Luca Donazzan

**Affiliations:** 1 Division of Cardiology, Department of Medicine, University of Verona, 37126 Verona, Italy; 2 Department of Cardiology, San Maurizio Hospital, 39100 Bolzano, Italy; 3 Department of Radiology, San Maurizio Hospital, 39100 Bolzano, Italy

**Keywords:** aortic stenosis, transcatheter aortic valve replacement, quality of life, Toronto Aortic Stenosis Quality of Life (TASQ) Questionnaire

## Abstract

Background: Sarcopenia is the core component of frailty; however, its role in patients undergoing transcatheter aortic valve replacement (TAVR) is a matter of debate. The Toronto Aortic Stenosis Quality of Life Questionnaire (TASQ) is a validated instrument for assessing quality of life (QoL) in patients with severe aortic stenosis (AS). Aims: We aim to evaluate the QoL of sarcopenic and non-sarcopenic patients with severe AS undergoing TAVR. Methods: TASQ was prospectively administered to patients undergoing TAVR. All patients completed the TASQ before TAVR and at a 3-month follow-up. The study population was divided in two groups according to sarcopenic status. The primary endpoint was the TASQ score in the sarcopenic and non-sarcopenic cohorts. Results: In total, 99 patients were eligible for the analysis. In both sarcopenic (*n* = 56) and non-sarcopenic (*n* = 43) cohorts, significant changes were observed in the overall TASQ score and in all but one (i.e., health expectations) of the individual domains (*p* < 0.01). Sarcopenic and non-sarcopenic patients showed substantial improvements across TASQ subscores. The mean change in overall TASQ score at three months revealed a significant improvement in both cohorts (*p* < 0.01). Health expectations worsened in sarcopenic patients at the 3-month follow-up (*p* = 0.06). Conclusions: The TASQ questionnaire revealed changes in QoL after TAVR, regardless of patients’ sarcopenic status. Health status improved substantially in both sarcopenic and non-sarcopenic patients following TAVR. Lack of improvement in health expectations seems to depend on patients’ expectations regarding the procedure and specific aspects in the evaluation of the outcome.

## 1. Introduction

Aortic valve stenosis (AS) is the most common type of valvular heart disease in developed countries and has an increasing prevalence worldwide due to ageing populations. Transcatheter aortic valve replacement (TAVR) is currently recommended for patients with severe symptomatic AS aged 75 years or above. This minimally invasive interventional aortic valve treatment reduces mortality at two years and improves quality of life mainly by reducing dyspnea [1,2]. However, 15% to 30% of patients reported no benefit from the intervention, experiencing no improvement in quality of life after TAVR [2,3]. In particular, geriatric syndromes seem to be strongly associated with less effective symptoms relief [4]. In this context, sarcopenia is the biological substrate of geriatric syndromes. Similar to AS, sarcopenia occurs as an age-related process in older people and is associated with serious health consequences in terms of frailty, disability, morbidity, and mortality [5]. The impact of sarcopenia on increased mortality, rehospitalization, or high-resource utilization after TAVR is still debated, with different studies giving diverging results [6,7,8]. Furthermore, current data regarding the impact of sarcopenia on the quality of life (QoL) of patients undergoing TAVR are sparse.

In this view, we performed a prospective study based on the Toronto Aortic Stenosis Quality of Life Questionnaire (TASQ) to evaluate health status outcomes [9,10] in objectively defined sarcopenic patients with severe symptomatic AS treated with TAVR as compared with non-sarcopenic patients.

## 2. Materials and Methods

### 2.1. Study Population

Consecutive patients aged 75 years or more with severe AS who underwent TAVR at the San Maurizio Hospital, Bolzano, Italy, in the context of an executive collaboration with the heart valve center of the University of Verona (Verona, Italy). Patients were recruited between October 2020 and May 2022. All patients underwent TAVR using either the SAPIEN (SAPIEN, SAPIEN XT, or SAPIEN 3; Edwards Lifesciences, Irvine, CA, USA) or the CoreValve^®^ (CoreValve^®^, EvolutTM R, or EvolutPRO Plus; Medtronic, Minneapolis, MN, USA) prosthesis. TAVR procedures were performed following the indications of the most recent consensus papers [11]. All patients provided signed informed consent before the procedure, in accordance with the ethical principles for medical research involving human subjects established by the Declaration of Helsinki, protecting the privacy of all participants, as well as the confidentiality of their personal information.

### 2.2. Clinical and Laboratory Measurements

Collected baseline data included age, gender, diabetes mellitus status, and New York Heart Association (NYHA) heart failure information, as well as data on history of moderate to severe pulmonary disease, atrial fibrillation, stroke, peripheral arterial disease, myocardial infarction, percutaneous coronary angioplasty, and coronary artery bypass grafting. Baseline echocardiography was performed in all patients before the TAVR procedure according to the guidelines of the European and American Society of Echocardiography [12].

### 2.3. Measurement of Quality of Life

We evaluated health status outcomes by using the TASQ (Supplementary Data S1), which is a 16-item health-specific QoL survey consisting of 5 domains addressing the physical, emotional, and social factors associated with AS disease. The scoring of the TASQ is based on a consistent 7-point scale for each of the 16 questions, with response options ranging from “not very much” to “very much”. Reversed scoring (except for question 16) is used so that increasing values are consistent with better QoL. Patients were required to complete the TASQ prior to the intervention (baseline) and at 3-month follow-up.

### 2.4. Measurement of Abdominal Muscle Mass

Abdominal computed tomography (CT) scan at the level of the L3 vertebra is the gold standard for non-invasive measurement of muscle mass [13]. Abdominal muscle mass measured by CT scan of the L3 vertebra is a good indicator of total body muscle mass and lower values are associated with adverse outcomes in different patient populations (Supplementary Figure S1) [13,14]. Muscle density was defined as densities from −29 to +150 Hounsfield units (HU), and skeletal muscle index was calculated by normalizing skeletal muscle area by height (m^2^) and reported as cm^2^/m^2^ [15]. The measurement of muscle mass obtained from pre-TAVR CT scan was carried out by an expert radiologist (M.B.) using a validated semi-automated software system for body composition [16].

### 2.5. Definitions

Glomerular filtration rate (GFR) was estimated using the Cockcroft–Gault equation formula. Chronic kidney disease was defined as stage 3, 4, or 5 chronic renal failure (estimated GFR < 60 mL/min/1.73 m^2^). The Society of Thoracic Surgeons (STS) risk score and EUROSCORE II were calculated for each patient according to published guidelines [17]. Sarcopenia was defined as skeletal muscle index values <55.4 cm^2^/m^2^ in males and <38.9 cm^2^/m^2^ in females. These gender-specific cut-off values were used in a previously published TAVR study [7,18].

### 2.6. Statistical Analysis

The primary endpoint was the TASQ score. The primary analysis evaluated health status in the sarcopenic and non-sarcopenic patients. Mean changes in health status scores were compared with baseline within each treatment group using paired *t*-tests. For the analyses, clinical categories were defined as worsening (decrease of >5% vs. baseline), no change (change of between −5% and 5%), slight improving (increase of >5–10% vs. baseline), moderate improving (increase of 10–20%), and substantial improving (increase of >20%), as already performed previously [9]. Baseline characteristics were compared between the cohorts using two-tailed *t*-tests for continuous variables and Fisher’s exact test for categorical variables (*p* values < 0.05 were regarded as statistically significant). Statistical analysis was performed using SPSS V.28.0.0.

## 3. Results

In total, 99 patients (50 males (50.5%)) underwent TAVR and completed the 3-month follow-up period. The mean age of the study population was 81.6 ± 4.3 years. Sarcopenia was prevalent in our study population (56 patients (56.6%)).

Sarcopenic patients were slightly older (82.5 ± 3.8 years vs. 80.4 ± 4.6 years, *p* < 0.01) and had lower body mass index (24.1 ± 4.4 kg/m^2^ vs. 27.6 ± 4.2 kg/m^2^, *p* < 0.01). Mean skeletal muscle index was 42.6 ± 9.5 cm^2^/m^2^ in sarcopenic and 51.2 ± 9.1 cm^2^/m^2^ in non-sarcopenic individuals (*p* < 0.01). Clinical presentation of patients with AS was not different between the two cohorts (Table 1).

It is worth noting that the presence of CAD was higher in the sarcopenic cohort when compared with the non-sarcopenic cohort (58.2% vs. 34.9%; *p* = 0.03); however, no difference was found in the prevalence of previous revascularization. Echocardiographic findings were similar in the two cohorts, although the mean aortic valve gradient was higher in non-sarcopenic patients when compared with sarcopenic individuals (51.4 ± 13.6 mmHg vs. 45.0 ± 11.6 mmHg; *p* = 0.01). Most other variables, including risk scores, were not different between the two groups (Table 1). There were no significant differences between the groups as far as baseline TASQ score was concerned (Table 2).

**Figure 1:**
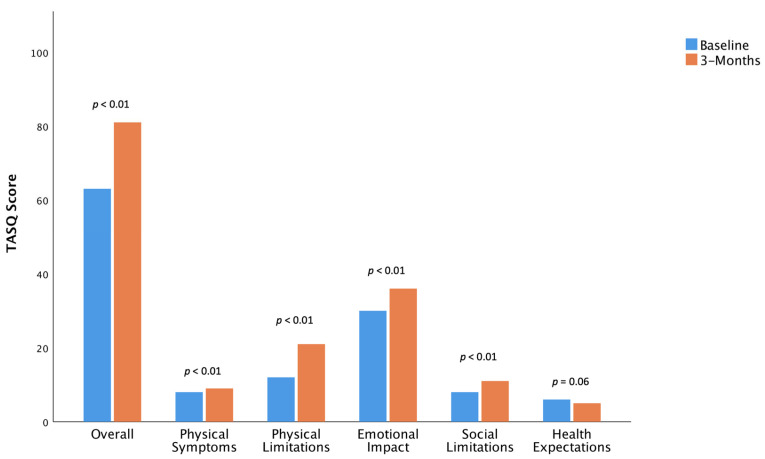
Absolute modification of TASQ score in sarcopenic patients at 3-month follow-up compared with baseline.

**Figure 2:**
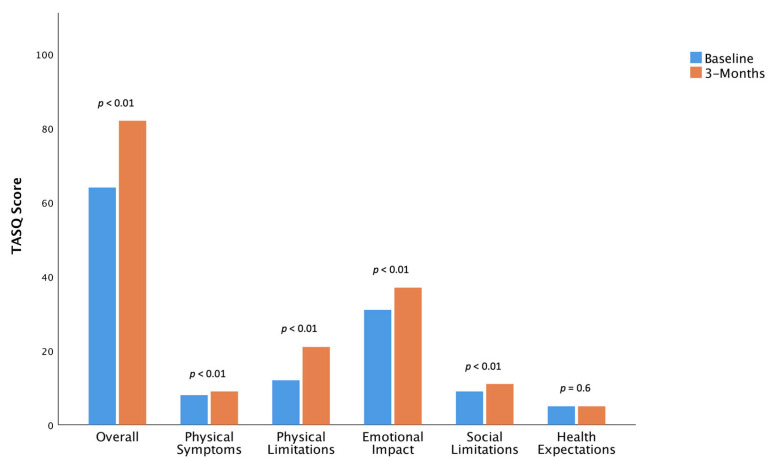
Absolute modification of TASQ score in non-sarcopenic patients at 3-month follow-up compared with baseline.

The absolute modification of the TASQ score in sarcopenic and non-sarcopenic patients at the 3-month follow-up compared with the baseline is reported in Figure 1 and Figure 2, respectively.

**Figure 3:**
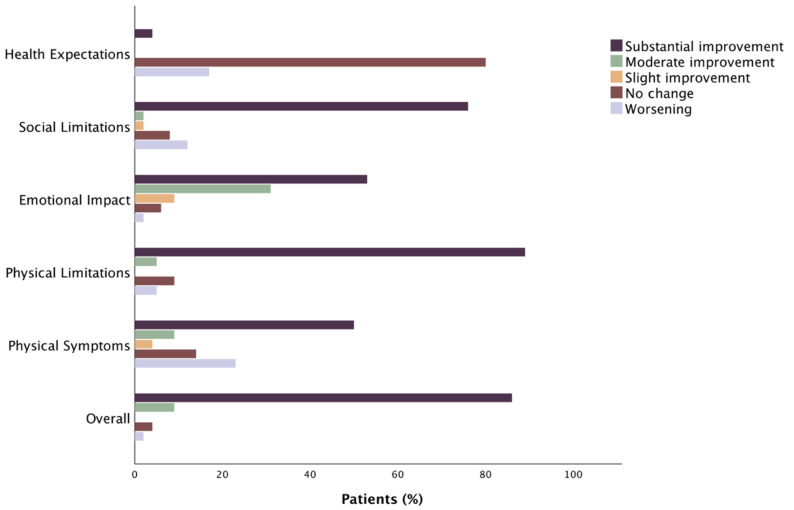
Proportion of sarcopenic patients who underwent TAVR achieving specific levels of clinically relevant change in TASQ Score.

**Figure 4:**
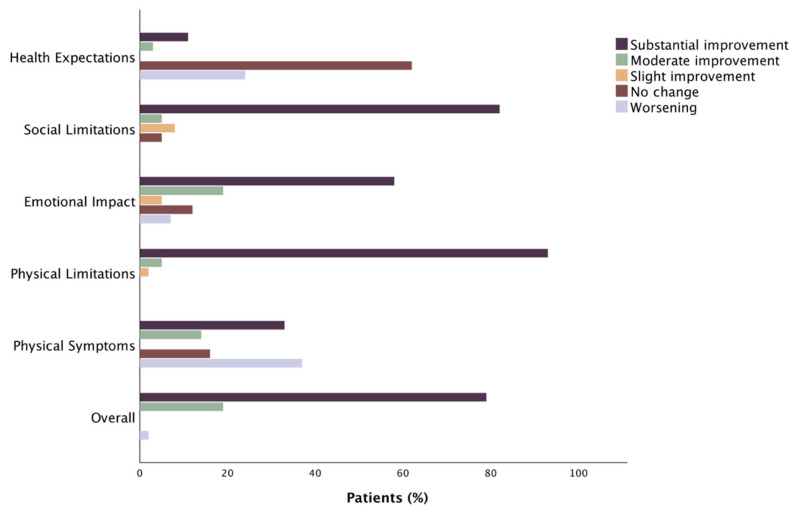
Proportion of non-sarcopenic patients who underwent TAVR achieving specific levels of clinically relevant change in TASQ Score.

Proportion of sarcopenic and non-sarcopenic patients who underwent TAVR achieving specific categorized levels of clinically relevant change in TASQ Score is depicted in Figure 3 and Figure 4, respectively.

In more detail, QoL improved after TAVR, as indicated by significant increases in the overall TASQ score in both sarcopenic and non-sarcopenic patients (+18.6 and +18.32, respectively, *p* < 0.01 for both) at three months (Table 3).

Improvement was achieved in 94.6% of sarcopenic and 97.7% of non-sarcopenic patients (Supplementary Tables S1 and S2).

Sarcopenic and non-sarcopenic patients showed a statistical overall improvement in “TASQ physical symptoms” (+1.67 and +1.33, respectively, *p* < 0.01 for both). A clinical improvement occurred in 62.5% of sarcopenic patients and in less than half of non-sarcopenic patients. A significant improvement in NYHA functional class was achieved in both sarcopenic and non-sarcopenic patients at 3-month follow-up (*p* < 0.05, Supplementary Figure S2).

There is agreement in the “TASQ physical limitations” domain between sarcopenic and non-sarcopenic patients, both in terms of statistical (+9.4 and +9.16, respectively, *p* < 0.01 for both) and clinical (in 94.7% and 97.7% of cases, respectively) improvements in their ability to perform daily activities. “TASQ social limitations” showed a significant improvement for both sarcopenic and non-sarcopenic patients (+5.87 and +6.33, respectively, *p* < 0.01 for both), with a clinical improvement reported in 80% of sarcopenic and 94.8% of non-sarcopenic patients. In the area of “TASQ emotional impact”, sarcopenic and non-sarcopenic patients showed a statistical improvement (+ 5.87 and + 6.33, respectively, *p* < 0.01 for both). The subanalysis by clinical level revealed no change or a worsening in TASQ emotional impact in 7.3% of sarcopenic patients and 18.6% of non-sarcopenic patients.

A trend towards a worsening in health expectations occurred in sarcopenic patients (−0.14; *p* = 0.06), whereas it showed no statistically significant changes (+0.05; *p* = 0.60) in non-sarcopenic patients at follow-up. The majority of sarcopenic patients presented a substantial absence in variability before and after the TAVR procedure.

## 4. Discussion

Sarcopenia is the core component of frailty, a frequent condition in patients undergoing TAVR. The functional impairment resulting from sarcopenic status may lead to decreased QoL prior to TAVR, and may also, theoretically, hamper the benefits after the intervention. Based on the principle that a good outcome has to take into account the disease as well as the illness status, we adopted the TASQ in order to address the knowledge gap of the currently available health-related QoL questionnaires. Indeed, the cornerstone randomized controlled trials on TAVR evaluated objective QoL measurements prior to and after TAVR using specific tools to heart failure symptoms [19,20,21,22,23,24]; however, they are not adequately designed to analyze the psychological implications of TAVR, especially regarding sarcopenic status.

Our study adds to the body of evidence about the TASQ, exploring whether CT-defined sarcopenia may affect short-term changes in QoL for patients who underwent TAVR.

First of all, it confirms an overall improvement in QoL after TAVR, as shown by significant increases in the overall TASQ score in both sarcopenic and non-sarcopenic patients at three months. This result reinforces the overall benefit of TAVR on QoL, as previously demonstrated using a variety of QoL tools [20,21,25,26] and, again, by TASQ [9,10,27].

Significant improvements occurred in four of the five TASQ domains: physical symptoms, physical limitations, emotional impact, and social limitations at the three-month follow-up.

Although both sarcopenic and non-sarcopenic patients showed a statistically significant overall improvement in physical symptoms, one-third of sarcopenic patients and more than half of non-sarcopenic patients reported no clinical changes or a worsened status after TAVR. These results emphasize the fact that there is only a proportion of patients—irrespective of sarcopenic status—who experience a benefit in reported physical symptoms after TAVR; however, this enhancement may explain the overall improvement observed in terms of statistical significance. A lack of changes or worsening in physical symptoms in non-sarcopenic patients may be driven by the large proportion of female patients, who are twice as likely as men to experience the relevant anxiety. This psychological condition may lead to an underrating of the heart health investigated in “TASQ physical symptoms”.

Sarcopenic and non-sarcopenic patients reported improvements in their ability to perform personally relevant activities rather than just their symptoms (such as walking without resting, doing daily chores). This observation, taken together with the agreement of both the absolute and clinically classified results on the “TASQ physical limitations” domain (Figure 1, Figure 2, Figure 3 and Figure 4), suggests that patients likely had an improvement in their physical limitations even though they continued to report the symptom “dyspnea”. In this view, TAVR seems to allow patients to better tolerate dyspnea during their daily chores in both sarcopenic and non-sarcopenic cohorts.

Importantly, the TASQ does not focus predominantly or specifically on physical symptoms or treatment issues but takes instead into account the social and emotional aspects of QoL, which in turn can hamper normal daily activities and social functioning. In this scenario, the results reported for the domain of “TASQ social limitations” reinforce the above speculation: although dyspnea remains a patient-reported symptom, TAVR allowed sarcopenic and non-sarcopenic patients to better tolerate this symptom. In fact, our patients reported a reduction in the significant limitations to their social lives (attending social events, meeting friends and family).

Furthermore, emotional status and overall emotional effects are also of great importance to QoL: this aspect is specifically investigated in a different TASQ domain. The “TASQ emotional impact” domain analyzed the daily anxiety concerning cardiac events, demonstrating a significantly reduced degree of worry about having a heart attack and about financial and family problems after TAVR. The subanalysis by clinical level revealed that the proportion of patients who experienced no change or saw a worsening in “TASQ emotional impact” was more than double in non-sarcopenic versus sarcopenic patients. This observation may suggest a higher baseline sensitivity to emotional changes in sarcopenic subjects that may report improvement more frequently after TAVR than non-sarcopenic patients, at least in the short term. More importantly, these results underline the need for support and attention by health professionals and caregivers to patients’ emotional wellbeing. Although ambitious, this goal should be pursued by delivering dedicated and personalized information, education, reassurance about the therapeutic strategy and, if needed, psychological support.

It is worth noting that we identified a trend towards a worsening in health expectations in the analyzed cohort. The majority of sarcopenic patients presented a substantial absence of variability before and after the TAVR procedure, although the statistical analysis showed an overall trend towards a worsening of life expectancy in these patients. The significantly older age of sarcopenic patients undergoing TAVR may have favored a reduction in the hope of future wellbeing, confirming previous results that observed how people with sarcopenia expect to live a higher proportion of years with disabilities [28]. To further confirm this point, no changes were found before and after the procedure in non-sarcopenic patients. Again, sarcopenia probably causes patients to develop a more significant emotional involvement than non-sarcopenic patients.

Taken together, the findings of our study reinforce the acknowledged role of patient expectations and perceptions on the impact of post-procedural outcomes, including QoL, [29,30] irrespective of their sarcopenic status.

### Strengths and Limitations

Our cohort of patients was small and the TASQ was only used to capture changes in QoL at a 3-month follow-up. Recruitment was aimed at facilitating an equal distribution of patient numbers across different languages (Italian and German). It will be therefore important to confirm the findings of this study in other patient groups. It would have been ideal to adjust for cognitive status as a confounding variable; however, this was not assessed in this study. Given the cross-sectional nature of the present study, the causal relationship between sarcopenia and quality of life could not be established.

## 5. Conclusions

Objectively defined sarcopenia did not hinder changes in QoL among patients with severe AS who were treated with TAVR. In fact, health status improved substantially in both sarcopenic and non-sarcopenic subjects after TAVR. The improvements in reported in the TASQ are statistically significant but occurred only in a limited number of patients, irrespective of their sarcopenic condition. A lack of improvement mostly involves patients’ health expectations, and may greatly depend on the patients’ unrealistic hopes regarding procedural effects, as well as the specific aspects involved in a personal evaluation of the outcome.

## Figures and Tables

**Table 1:** Baseline characteristics of the study population pre-TAVR.

	Sarcopenic (*n* = 56)	Non-Sarcopenic (*n* = 43)	*p*-Value
Demographics			
Age, years	82.5 ± 3.8	80.4 ± 4.6	<0.01
Men, *n* (%)	34 (60.7%)	16 (37.2%)	0.02
Body mass index, kg/m^2^	24.1 ± 4.4	27.6 ± 4.2	<0.01
Skeletal muscle index, cm^2^/m^2^	42.6 ± 9.5	51.2 ± 9.1	<0.01
Cardiovascular risk factor			
Hypertension, *n* (%)	48 (85.7%)	35 (81.4)	0.59
Hypercholesterolemia, *n* (%)	35 (60.5%)	26 (62.5)	0.83
Moderate or severe pulmonary disease, *n* (%)	7 (12.5%)	3 (7%)	0.50
Diabetes mellitus, *n* (%)	12 (21.4%)	10 (23.3%)	0.99
Medical history			
Prior myocardial infarction, *n* (%)	8 (14.3%)	4 (9.3%)	0.54
Prior coronary revascularization, *n* (%)	11 (19.6%)	6 (14.0%)	0.59
Prior stroke, *n* (%)	4 (7.1%)	5 (11.6%)	0.49
Coronary artery disease, *n* (%)	32 (58.2%)	15 (34.9%)	0.03
Peripheral artery disease, *n* (%)	21 (37.5%)	16 (37.2%)	0.99
Atrial fibrillation/flutter, *n* (%)	11 (19.7%)	13 (30.2%)	0.34
Echocardiographic findings			
Aortic valve area index, cm^2^/m^2^	0.41 ± 0.13	0.39 ± 0.15	0.11
Mean aortic valve gradient, mmHg	45 ± 11.6	51.4 ± 13.6	0.01
Moderate or severe mitral regurgitation, *n* (%)	9 (20.9%)	10 (17.9%)	0.79
Moderate or severe tricuspid regurgitation, *n* (%)	7 (12.5%)	6 (14.0%)	0.99
Left ventricular ejection fraction, *n* (%)	58.9 ± 10.2	57.7 ± 7.6	0.55
Admission and procedural characteristics			
Exertional angina, *n* (%)	18 (32.1)	12 (27.9)	0.66
Acute heart failure, *n* (%)	4 (7.1%)	4 (9.3%)	0.72
NYHA III–IV, *n* (%)	20 (35.7%)	19 (44.2%)	0.41
Syncope, *n* (%)	7 (12.5%)	10 (23.3%)	0.18
Serum hemoglobin, mg/dL	13 ± 1.4	12.6 ± 1.5	0.28
Serum creatinine, mg/dL	1.12 ± 0.6	1.12 ± 0.3	0.98
Chronic kidney disease, *n* (%)	40 (71.4%)	32 (74.4%)	0.82
Porcelain aorta, *n* (%)	2 (3.6%)	5 (11.6%)	0.23
STS risk score	3.37 ± 1.6	3.42 ± 2.2	0.90
Euroscore II	3.79 ± 2.7	3.71 ± 2.9	0.89
Valve type: Edwards	17 (30.4%)	13 (30.2%)	0.90
Valve type: Medtronic	39 (69.6%)	30 (69.8%)	0.90
Non-femoral access route, *n* (%)	2 (3.6%)	0 (0%)	0.50

NYHA = New York Heart Association; STS Score = Society of Thoracic Surgeons Score.

**Table 2:** Baseline TASQ score of the study population pre-TAVR.

	Sarcopenic (*n* = 56)	Non-Sarcopenic (*n* = 43)	*p*-Value
TASQ overall summary	62.9 ± 10.1	63.9 ± 9.2	0.60
TASQ physical symptoms	7.5 ± 2.5	7.4 ± 2.6	0.99
TASQ physical limitations	11.5 ± 3.8	12 ± 3.7	0.56
TASQ emotional impact	30.1 ± 6.5	30.5 ± 5.7	0.97
TASQ social limitations	8.3 ± 1.7	8.5 ± 1.6	0.64
TASQ health expectations	5.6 ± 0.9	5.3 ± 0.7	0.13

TASQ = Toronto Aortic Stenosis Quality of Life Questionnaire.

**Table 3:** Within-group change in TASQ in sarcopenic vs. non-sarcopenic patients at three-month follow-up.

	Sarcopenic (*n* = 56)		Non-Sarcopenic (*n* = 43)	
	Paired Difference versus Baseline (95% CI)	*p*-Value	Paired Difference versus Baseline (95% CI)	*p*-Value
TASQ overall summary	18.6 (16.39 to 20.74)	<0.01	18.32 (15.06 to 21.58)	<0.01
TASQ physical symptoms	1.67 (0.83 to 2.51)	<0.01	1.33 (0.35 to 2.31)	<0.01
TASQ physical limitations	9.4 (8.32 to 10.48)	<0.01	9.16 (8.08 to 10.23)	<0.01
TASQ emotional impact	5.87 (4.79 to 6.95)	<0.01	6.33 (4.16 to 8.519	<0.01
TASQ social limitations	2.5 (1.78 to 3.23)	<0.01	2.48 (2.01 to 2.96)	<0.01
TASQ health expectations	−0.14 (−0.29 to 0.005)	0.06	0.05 (−0.16 to 0.26)	0.60

TASQ = Toronto Aortic Stenosis Quality of Life Questionnaire.

## Data Availability

Data are available from the authors upon reasonable request.

## Supplementary Materials

The following supporting information can be downloaded at: https://www.mdpi.com/article/10.3390/jcm12052078/s1, Figure S1. CT scans of a sarcopenic and a non-sarcopenic patient. Figure S2. New York Heart Association (NYHA) class in sarcopenic and non-sarcopenic patients prior to TAVR and at 3 months after TAVR. Table S1. Proportion of sarcopenic patients (*n* = 56) who underwent TAVR achieving specific levels of clinically relevant change in health status. Table S2. Proportion of non-sarcopenic patients (*n* = 43) who underwent TAVR achieving specific levels of clinically relevant change in health status. Data S1. Example of the Toronto Aortic Stenosis Quality of Life Questionnaire (TASQ).

## References

[1] Reardon M.J., van Mieghem N.M., Popma J.J., Kleiman N.S., Søndergaard L., Mumtaz M., Adams D.H., Deeb G.M., Maini B., Gada H. (2017). Surgical or Transcatheter Aortic-Valve Replacement in Intermediate-Risk Patients. N. Engl. J. Med..

[2] Zahn R., Werner N., Gerckens U., Linke A., Sievert H., Kahlert P., Hambrecht R., Sack S., Abdel-Wahab M., Hoffmann E. (2017). Five-year follow-up after transcatheter aortic valve implantation for symptomatic aortic stenosis. Heart.

[3] Deeb G.M., Reardon M.J., Chetcuti S., Patel H.J., Grossman P.M., Yakubov S.J., Kleiman N.S., Coselli J.S., Gleason T.G., Lee J.S. (2016). 3-Year Outcomes in High-Risk Patients Who Underwent Surgical or Transcatheter Aortic Valve Replacement. J. Am. Coll. Cardiol..

[4] Afilalo J., Lauck S., Kim D.H., Lefèvre T., Piazza N., Lachapelle K., Martucci G., Lamy A., Labinaz M., Peterson M.D. (2017). Frailty in Older Adults Undergoing Aortic Valve Replacement: The FRAILTY-AVR Study. J. Am. Coll. Cardiol..

[5] Marty E., Liu Y., Samuel A., Or O., Lane J. (2017). A review of sarcopenia: Enhancing awareness of an increasingly prevalent disease. Bone.

[6] Mamane S., Mullie L., Lok Ok Choo W., Piazza N., Martucci G., Morais J.A., Kim D.H., Lauck S., Webb J.G., Afilalo J. (2019). Sarcopenia in Older Adults Undergoing Transcatheter Aortic Valve Replacement. J. Am. Coll. Cardiol..

[7] Heidari B., Al-Hijji M.A., Moynagh M.R., Takahashi N., Welle G., Eleid M., Singh M., Gulati R., Rihal C.S., Lerman A. (2019). Transcatheter aortic valve replacement outcomes in patients with sarcopaenia. EuroIntervention.

[8] Tokuda T., Yamamoto M., Kagase A., Koyama Y., Otsuka T., Tada N., Naganuma T., Araki M., Yamanaka F., Shirai S. (2020). Importance of combined assessment of skeletal muscle mass and density by computed tomography in predicting clinical outcomes after transcatheter aortic valve replacement. Int. J. Cardiovasc. Imaging.

[9] Kennon S., Styra R., Bonaros N., Stastny L., Romano M., Lefevre T., Di Mario C., Stefàno P., Ribichini F.L., Himbert D. (2021). Quality of life after transcatheter or surgical aortic valve replacement using the Toronto Aortic Stenosis Quality of Life Questionnaire. Open Heart.

[10] Frank D., Kennon S., Bonaros N. (2021). Aortic valve replacement: Validation of the Toronto aortic stenosis quality of life questionnaire. ESC Heart Fail..

[11] Otto C.M., Kumbhani D.J., Alexander K.P., Calhoon J.H., Desai M.Y., Kaul S., Li J.C. (2017). 2017 ACC Expert Consensus Decision Pathway for Transcatheter Aortic Valve Replacement in the Management of Adults With Aortic Stenosis: A Report of the American College of Cardiology Task Force on Clinical Expert Consensus Documents. J. Am. Coll. Cardiol..

[12] Lang R.M., Badano L.P., Victor M.A., Afilalo J., Armstrong A., Ernande L., Flachskampf F.A., Foster E., Goldstein S.A., Kuznetsova T. (2015). Recommendations for cardiac chamber quantification by echocardiography in adults: An update from the American Society of Echocardiography and the European Association of Cardiovascular Imaging. J. Am. Soc. Echocardiogr..

[13] Cruz-Jentoft A.J., Bahat G., Bauer J., Boirie Y., Bruyère O., Cederholm T., Cooper C., Landi F., Rolland Y., Sayer A.A. (2019). Sarcopenia: Revised European consensus on definition and diagnosis. Age Ageing.

[14] Fearon K., Strasser F., Anker S.D., Bosaeus I., Bruera E., Fainsinger R.L., Jatoi A., Loprinzi C., MacDonald N., Mantovani G. (2011). Definition and classification of cancer cachexia: An international consensus. Lancet Oncol..

[15] Derstine B.A., Holcombe S.A., Ross B.E., Wang N.C., Su G.L., Wang S.C. (2018). Skeletal muscle cutoff values for sarcopenia diagnosis using T10 to L5 measurements in a healthy US population. Sci. Rep..

[16] Irving B.A., Weltman J.Y., Brock D.W., Davis C.K., Gaesser G.A., Weltman A. (2007). NIH ImageJ and Slice-O-Matic computed tomography imaging software to quantify soft tissue. Obesity (Silver Spring).

[17] Vahanian A., Beyersdorf F., Praz F., Milojevic M., Baldus S., Bauersachs J., Capodanno D., Conradi L., De Bonis M., De Paulis R. (2022). 2021 ESC/EACTS Guidelines for the management of valvular heart disease. Eur. Heart J..

[18] Mok M., Allende R., Leipsic J., Altisent O.A.J., del Trigo M., Campelo-Parada F., DeLarochellière R., Dumont E., Doyle D., Côté M. (2016). Prognostic Value of Fat Mass and Skeletal Muscle Mass Determined by Computed Tomography in Patients Who Underwent Transcatheter Aortic Valve Implantation. Am. J. Cardiol..

[19] Kapadia S.R., Leon M.B., Makkar R.R., Tuzcu E.M., Svensson L.G., Kodali S., Webb J.G., Mack M.J., Douglas P.S., Thourani V.H. (2015). 5-year outcomes of transcatheter aortic valve replacement compared with standard treatment for patients with inoperable aortic stenosis (PARTNER 1): A randomised controlled trial. Lancet.

[20] Osnabrugge R.L., Arnold S.V., Reynolds M.R., Magnuson E.A., Wang K., Gaudiani V.A., Stoler R.C., Burdon T.A., Kleiman N., Reardon M.J. (2015). Health status after transcatheter aortic valve replacement in patients at extreme surgical risk: Results from the CoreValve U.S. Trial. JACC Cardiovasc. Interv..

[21] Reynolds M.R., Magnuson E.A., Wang K., Thourani V.H., Williams M., Zajarias A., Rihal C.S., Brown D.L., Smith C.R., Leon M.B. (2012). Health-related quality of life after transcatheter or surgical aortic valve replacement in high-risk patients with severe aortic stenosis: Results from the PARTNER (Placement of AoRTic TraNscathetER Valve) Trial (Cohort A). J. Am. Coll. Cardiol..

[22] Adams D.H., Popma J.J., Reardon M.J., Yakubov S.J., Coselli J.S., Deeb G.M., Gleason T.G., Buchbinder M., Hermiller J., Kleiman N.S. (2014). Transcatheter aortic-valve replacement with a self-expanding prosthesis. N. Engl. J. Med..

[23] Green C.P., Porter C.B., Bresnahan D.R., Spertus J.A. (2000). Development and evaluation of the Kansas City Cardiomyopathy Questionnaire: A new health status measure for heart failure. J. Am. Coll. Cardiol..

[24] Damluji A.A., Rodriguez G., Noel T., Davis L., Dahya V., Tehrani B., Epps K., Sherwood M., Sarin E., Walston J. (2020). Sarcopenia and health-related quality of life in older adults after transcatheter aortic valve replacement. Am. Heart J..

[25] Reynolds M.R., Magnuson E.A., Lei Y., Leon M.B., Smith C.R., Svensson L.G., Webb J.G., Babaliaros V.C., Bowers B.S., Fearon W.F. (2011). Health-Related Quality of Life After Transcatheter Aortic Valve Replacement in Inoperable Patients With Severe Aortic Stenosis. Circulation.

[26] Lauck S.B., Arnold S.V., Borregaard B., Sathananthan J., Humphries K.H., Baron S.J., Wijeysundera H.C., Asgar A., Welsh R., Velianou J.L. (2020). Very Early Changes in Quality of Life After Transcatheter Aortic Valve Replacement: Results From the 3M TAVR Trial. Cardiovasc. Revascularization Med..

[27] Frank D., Kennon S., Bonaros N. (2019). Trial protocol for the validation of the ‘Toronto Aortic Stenosis Quality of Life (TASQ) Questionnaire’ in patients undergoing surgical aortic valve replacement (SAVR) or transfemoral (TF) transcatheter aortic valve implantation (TAVI): The TASQ registry. Open Heart.

[28] Moreno X., Lera L., Márquez C., Albala C. (2022). Forecasting Healthy Life Expectancy Among Chilean Community-Dwelling Older Adults With and Without Sarcopenia. Front. Med..

[29] Waljee J., McGlinn E.P., Sears E.D., Chung K.C. (2014). Patient expectations and patient-reported outcomes in surgery: A systematic review. Surgery.

[30] Lunardi M., Kennedy C., Prabhakar A., Mylotte D. (2022). Transcatheter aortic valve replacement: When should we say no?. Open Heart.

