# Supplementary material for: Quality of Life after Transcatheter Aortic Valve Replacement in Sarcopenic Patients Using the Toronto Aortic Stenosis Quality of Life Questionnaire
Source: J Clin Med. 2023 Mar 6;12(5):2078. doi: 10.3390/jcm12052078 (PMC10003854; doi:10.3390/jcm12052078)
Supplement: Supplementary file 1 [file jcm-12-02078-s001.zip › Supplementary Data S1.pdf]

Supplementary Data S1

Example of the Toronto Aortic Stenosis Quality of Life Questionnaire (TASQ)

This questionnaire helps us to better understand your heart problems, in particular aortic stenosis and how it has affected you. For each question, please circle the number that best describes your current health situation.

1. **ARE YOU SHORT OF BREATH?**

   NOT VERY MUCH 1 2 3 4 5 6 7 VERY MUCH

2. **ARE YOU WORRIED ABOUT HAVING A HEART ATTACK OR DYING?**

   NOT VERY MUCH 1 2 3 4 5 6 7 VERY MUCH

3. **DO YOUR HEART PROBLEMS INTERFERE WITH DOING DAILY CHORES?**

   NOT VERY MUCH 1 2 3 4 5 6 7 VERY MUCH

4. **DO YOUR HEART PROBLEMS INTERFERE WITH GOING OUT TO VISIT FRIENDS OR SOCIAL EVENTS?**

   NOT VERY MUCH 1 2 3 4 5 6 7 VERY MUCH

5. **DO YOUR HEART PROBLEMS INTERFERE WITH GOING OUT TO VISIT FAMILY?**

   NOT VERY MUCH 1 2 3 4 5 6 7 VERY MUCH

6. **DO YOUR HEART PROBLEMS INTERFERE WITH BEING ABLE TO WALK WITHOUT RESTING?**

   NOT VERY MUCH 1 2 3 4 5 6 7 VERY MUCH

7. **ARE YOU SHORT OF BREATH OR VERY TIRED WHEN EXERCISING?**

   NOT VERY MUCH 1 2 3 4 5 6 7 VERY MUCH

8. **ARE YOU FRUSTRATED ABOUT HAVING TO STAY IN OR GO TO THE HOSPITAL BECAUSE OF HEART PROBLEMS?**

   NOT VERY MUCH 1 2 3 4 5 6 7 VERY MUCH

9. **ARE YOU FEELING DISCOURAGED ABOUT BEING VERY TIRED?**

   NOT VERY MUCH 1 2 3 4 5 6 7 VERY MUCH

10. **ARE YOU WORRIED ABOUT WHAT WILL HAPPEN TO YOUR FAMILY IF YOU DON'T GET BETTER?**

    NOT VERY MUCH 1 2 3 4 5 6 7 VERY MUCH

11. **ARE YOU WORRIED ABOUT WHAT WILL HAPPEN FINANCIALLY?**

    NOT VERY MUCH 1 2 3 4 5 6 7 VERY MUCH

12. **DO YOU FEEL YOU ARE UNABLE TO MAKE PLANS FOR THE FUTURE?**

    NOT VERY MUCH 1 2 3 4 5 6 7 VERY MUCH

13. **IS YOUR ENJOYMENT OF LIFE LIMITED BY YOUR HEART HEALTH PROBLEMS?**

NOT VERY MUCH 1 2 3 4 5 6 7 VERY MUCH

14. **I WOULD RATE MY OVERALL HEART HEALTH TO BE:**

Excellent Very good Good Fair Poor Very Poor Terrible

15. **COMPARED TO TWO MONTHS AGO, HOW WOULD YOU RATE YOUR ABILITY TO DO THINGS?**

Very much better than two months ago

Much better than two months ago

Somewhat better than two months ago

About the same

Somewhat worse than two months ago

Much worse than two months ago

Very much worse than two months ago

16. **COMPARED TO TWO MONTHS AGO, DO YOU FEEL HOPEFUL THAT YOUR HEALTH WILL IMPROVE?**

NOT VERY MUCH 1 2 3 4 5 6 7 VERY MUCH
