# Supplementary material for: Quality of Life after Transcatheter Aortic Valve Replacement in Sarcopenic Patients Using the Toronto Aortic Stenosis Quality of Life Questionnaire
Source: J Clin Med. 2023 Mar 6;12(5):2078. doi: 10.3390/jcm12052078 (PMC10003854; doi:10.3390/jcm12052078)
Supplement: Supplementary file 1 [file jcm-12-02078-s001.zip › Supplementary Tables and Figures.pdf]

Supplementary Materials

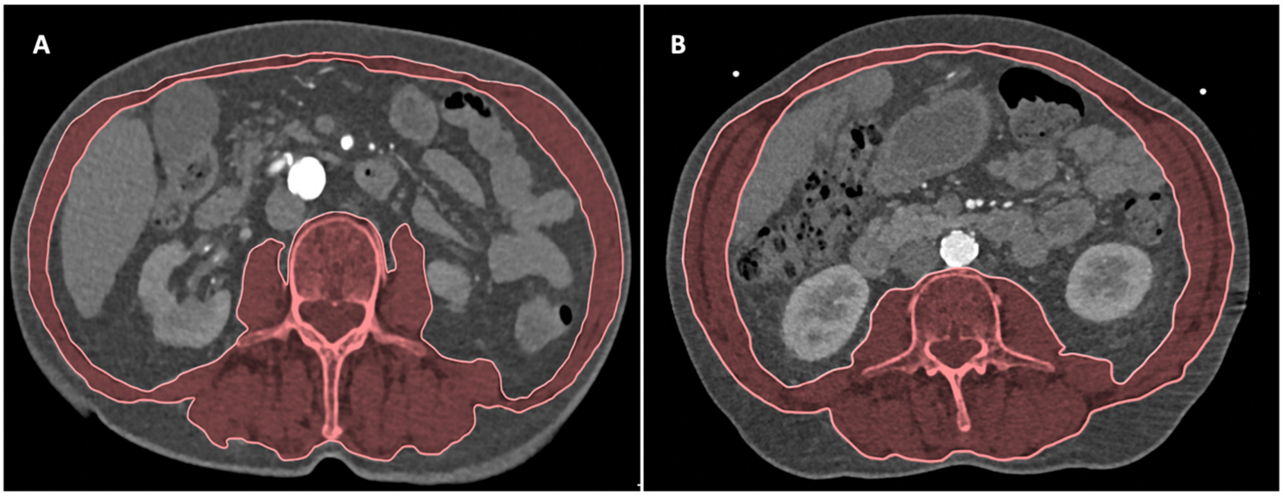

Figure S1. CT scans of a sarcopenic and a non-sarcopenic patient. (**A**) Sarcopenic patient. (**B**) Non-sarcopenic patient. Sarcopenic status was defined according to skeletal muscle index values <55.4 cm^2/m^2 in males and <38.9 cm^2/m^2 in females.

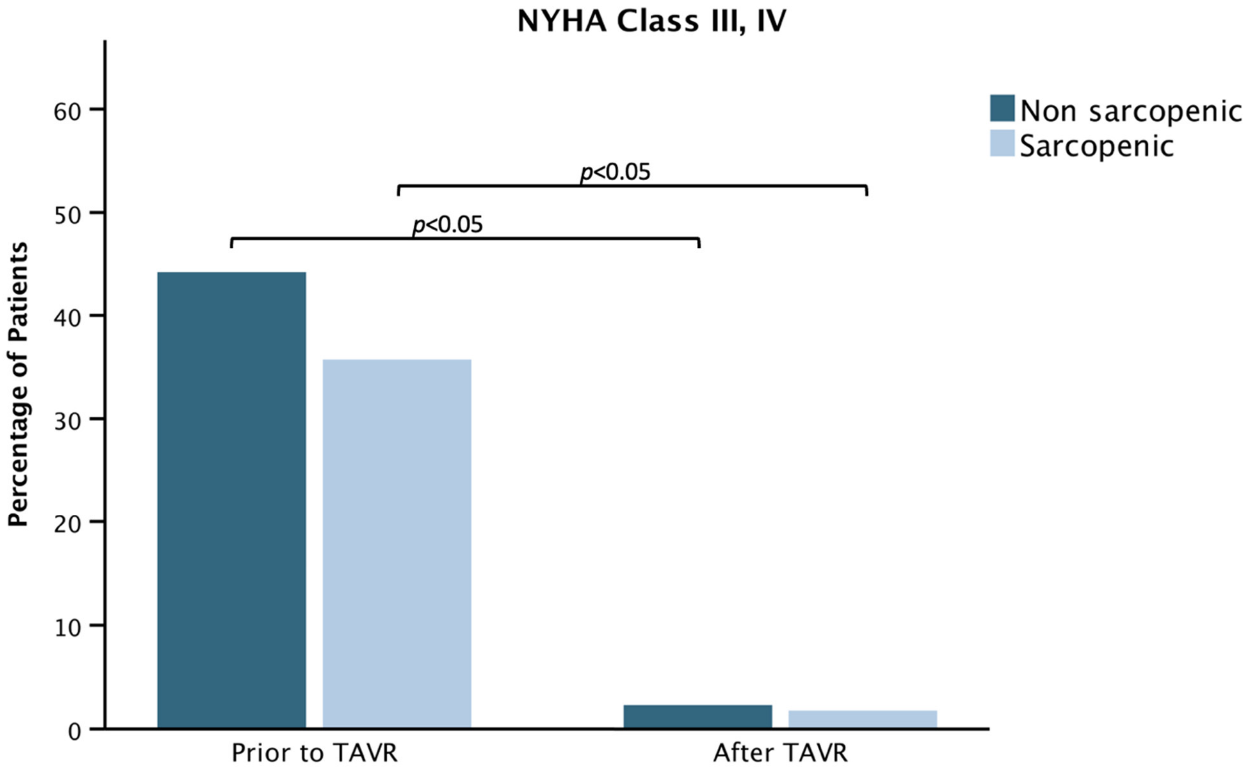

Figure S2. New York Heart Association (NYHA) class in sarcopenic and non-sarcopenic patients prior to TAVR and at 3 months after TAVR.

Table S1. Proportion of sarcopenic patients (*n* = 56) who underwent TAVR achieving specific levels of clinically relevant change in health status.

	Physical Symptoms	Physical Limitations	Emotional Impact	Social Limitations	Health Expectations	Overall Summary
Substantially improved	50.0%	89.3%	52.7%	76%	3.7%	85.7%
Moderately improved	8.9%	5.4%	30.9%	2%	0%	8.9%
Slightly improved	3.6%	0.0%	9.1%	2%	0%	0.0%
No change	14.3%	0.0%	5.5%	8%	79.6%	3.6%
Worse	23.2%	5.4%	1.8%	12%	16.7%	1.8%

Table S2. Proportion of non-sarcopenic patients (*n* = 43) who underwent TAVR achieving specific levels of clinically relevant change in health status.

	Physical Symptoms	Physical Limitations	Emotional Impact	Social Limitations	Health Expectations	Overall Summary
Substantially improved	32.6%	93.0%	58.1%	81.6%	10.8%	79.1%
Moderately improved	14.0%	4.7%	18.6%	5.3%	2.7%	18.6%
Slightly improved	0.0%	2.3%	4.7%	7.9%	0.0%	0.0%
No change	716.3%	0.0%	11.6%	5.3%	62.2%	0.0%
Worse	37.2	0.0%	7.0%	0.0%	24.3%	2.3%
